# Current News

**Published:** 1891-08

**Authors:** 


### Current News.

---

### MEETING OF COMMITTEES ON DENTAL PATENTS.

THERE will be a meeting held at the Town Hall, Saratoga, Monday evening, August 3, at eight o'clock, of all the committees appointed by the different dental societies all over the United States, for the purpose of organizing and taking some action towards the prevention of the further issuing of patents upon operations in the mouth. It is hoped that every society will see to it that they are represented at this meeting. Remember it will be held the Monday evening preceding the meeting of the American Dental Association.

S. C. G. WATKINS, *Chairman,*
W. L. FISH, *Secretary,*
*Of New Jersey Committee.*

---

### HARVARD DENTAL ALUMNI ASSOCIATION.

THE Twentieth Annual Meeting and Banquet of the Harvard Dental Alumni Association was held at the "Thorndike," Boston, June 23, 1891.

The meeting was the largest and most interesting ever held, about eighty of the Alumni being present.

CHARLES H. TAFT,
*Secretary.*

[The Constitution of the Harvard Dental Alumni Association was forwarded with the above. We regret not having room for it.—ED.]

---

ACCEPTED A PROFESSORSHIP.—W. C. Barrett, M.D., D.D.S., of Buffalo, N. Y., formerly editor of the *Independent Practitioner*, has accepted the professorship of dental anatomy and pathology in the Chicago College of Dental Surgery. He will enter upon his duties at the fall term, which begins in September. Dr. Barrett is one of the most distinguished members of his profession in the East. —*Western Paper.*

